# An exploration of Canadian government officials’ COVID-19 messages and the public’s reaction using social media data

**DOI:** 10.1371/journal.pone.0273153

**Published:** 2022-09-02

**Authors:** Amine Kada, Arbi Chouikh, Sehl Mellouli, Anupa J. Prashad, Sharon E. Straus, Christine Fahim

**Affiliations:** 1 Department of Management Information Systems, Faculty of Business Administration, Université Laval, Quebec, Canada; 2 Knowledge Translation Program, Li Ka Shing Knowledge Institute, Unity Health Toronto, Toronto, Canada; 3 IHPME, Dalla Lana School of Public Health, University of Toronto, Toronto, Canada; Universitá degli Studi di Milano, ITALY

## Abstract

Governments can use social media platforms such as Twitter to disseminate health information to the public, as evidenced during the COVID-19 pandemic [Pershad (2018)]. The purpose of this study is to gain a better understanding of Canadian government and public health officials’ use of Twitter as a dissemination platform during the pandemic and to explore the public’s engagement with and sentiment towards these messages. We examined the account data of 93 Canadian public health and government officials during the first wave of the pandemic in Canada (December 31, 2019 August 31, 2020). Our objectives were to: 1) determine the engagement rates of the public with Canadian federal and provincial/territorial governments and public health officials’ Twitter posts; 2) conduct a hashtag trend analysis to explore the Canadian public’s discourse related to the pandemic during this period; 3) provide insights on the public’s reaction to Canadian authorities’ tweets through sentiment analysis. To address these objectives, we extracted Twitter posts, replies, and associated metadata available during the study period in both English and French. Our results show that the public demonstrated increased engagement with federal officials’ Twitter accounts as compared to provincial/territorial accounts. For the hashtag trends analysis of the public discourse during the first wave of the pandemic, we observed a topic shift in the Canadian public discourse over time between the period prior to the first wave and the first wave of the pandemic. Additionally, we identified 11 sentiments expressed by the public when reacting to Canadian authorities’ tweets. This study illustrates the potential to leverage social media to understand public discourse during a pandemic. We suggest that routine analyses of such data by governments can provide governments and public health officials with real-time data on public sentiments during a public health emergency. These data can be used to better disseminate key messages to the public.

## 1. Introduction

The novel coronavirus (COVID-19, SARS-CoV-2) was first documented in December 2019 in Wuhan City, Hubei Province, China [2]. The virus spread rapidly around the world and by March 11, 2020, the World Health Organization declared the COVID-19 outbreak a pandemic [3]. Worldwide, as of June 20, 2021, a total of 178,433,920 cases of COVID-19 and 3,864,731 deaths were confirmed in 192 countries and regions [4], with cases surpassing those of the Middle East Respiratory Syndrome (MERS), the Severe Acute Respiratory Syndrome (SARS) and the previous H1N1 epidemics [5–7].

Unfortunately, the COVID-19 pandemic has led to difficult situations such as deaths, post-COVID psychological damage, or substantial pressure on the healthcare systems. It presents critical risks for individuals and the communities. This pandemic can be viewed as an unfortunate event that has caused direct harm to individuals [8]: it is a global catastrophe, it cannot be easily eradicated, and it brings new risks such as virus mutations that are unknown to the scientific community. As cases increased and community spread worsened, government and public health officials worldwide adopted measures to reduce transmission including handwashing [9], social distancing, and self-isolation following exposure [10]. In this context, governments needed to use all the required means to mitigate the risks associated with COVID-19. To this end, many officials turned to social media, in addition to traditional media sources (e.g., television, radio), as a platform to disseminate relevant health information to the public [11, 12]. By all these measures and actions, the objective of governments has been to reduce all risks that are related to the pandemic.

The purpose of our study is to provide insights on how Canadian federal and provincial/territorial governments and Canadian and provincial/territorial public health officials used Twitter as a platform to disseminate COVID-19 information and to determine the public’s engagement with and reaction to these messages. Specifically, our objectives are to 1) determine the engagement rates of the public with the Canadian federal and provincial government and public health officials’ Twitter posts; 2) assess hashtag trends and topic shift related to the Canadian public discourse on Twitter regarding COVID-19; 3) conduct an in-depth sentiment analysis of Twitter users’ responses to government and public health officials’ COVID-19-related Twitter posts.

The paper is organized as follows: Section 2 provides the theoretical background of the study, Section 3 describes the adopted methodology. Section 4 is devoted to the results that are discussed in Section 5. Section 6 concludes the paper.

## 2. Theoretical background

In this paper, we consider the COVID-19 pandemic as an unfortunate event that has caused direct harm to individuals including deaths, admission in intensive care for those with severe COVD-19, restrictions for people to travel, people losing their jobs, etc. In this context, governments should use all means available to mitigate risks associated with the COVID-19 pandemic. To this end, they need to communicate all the mitigation strategies to their citizens and make them aware of all faced risks. The importance of risk communication during public health emergencies has been consistently highlighted in the literature [13–16]. Risk communication describes any purposeful exchange of information about risks between interested parties (e.g., such as from a government organization to the public) [17]. In risk communication, there are three components to consider [18]: 1) the message features, which define the characteristics of the message to be shared with people, 2) the messengers, which are the entities that send the message, and 3) the audience, who are the people to whom the message features are sent. Based on these components, governments (who are the messengers) communicate to the public (who is the audience) on the COVID-19 pandemic (which is the message).

This communication can be conducted in different ways and should be a two-way communication [8]. In order not to fail, governments need to provide information to mitigate the risks and people perceptions of (and reactions to) these risks. To this end, governments turned to social media platforms since they allow this bidirectional communication between governments and people. In fact, in previous pandemic situations where social media has been used (such as the H1N1 outbreak and the Ebola outbreak), social media has been recognized as being an excellent vector to communicate with people [17, 19]. Specifically, successful risk communication strategies are built on the trust of public health officials [20]. Hence, it is very important for governments to provide people with the confidence in their preparedness to address the pandemic; they need to ensure that they are providing accurate information and that people are convinced by the measures put in place [21]. It then becomes interesting to investigate how governments used social media to disseminate messages to their citizens to better inform them about the risks that COVID-19 presents to society as a whole and to see how people reacted to these messages.

The study conducted in this paper takes place in Canada. Canada is a federal country where power is shared between the federal government and provinces/territories governments. There is a paucity of data describing how the public reacted to COVID-19-related public health messages provided by varying levels of government in Canada. The purpose of this study is therefore to 1) determine the engagement rates of the public with Canadian federal and provincial/territorial governments and public health officials’ Twitter posts; 2) conduct a hashtag trend analysis to explore the Canadian public’s discourse related to the pandemic during this period; 3) provide insights on the public’s reaction to Canadian authorities’ tweets through sentiment analysis.

## 3. Methods

### 3.1. Twitter as the target data platform

We chose Twitter as our research social media platform. Twitter is a microblogging and social networking platform on which registered users post and interact with 280-character messages known as *tweets*. We chose Twitter as the target research social media platform since it is one of the world’s largest social media platforms and allows users to disseminate real-time information to a wide audience [22]. Since its launch, Twitter has become an important channel for public communication [23]. Its usefulness, efficiency, and impact has particularly been demonstrated in the contexts of politics [24], natural disasters crises [25], brand communications [26], and everyday interpersonal exchanges [27]. Twitter has evolved as a key public health dissemination tool, as was observed during the COVID-19 pandemic. Furthermore, Twitter has been increasingly used to conduct research, as it allows for the study of large-scale, world-wide-web communications [28].

### 3.2. Conceptual framework

**Fig 1:**
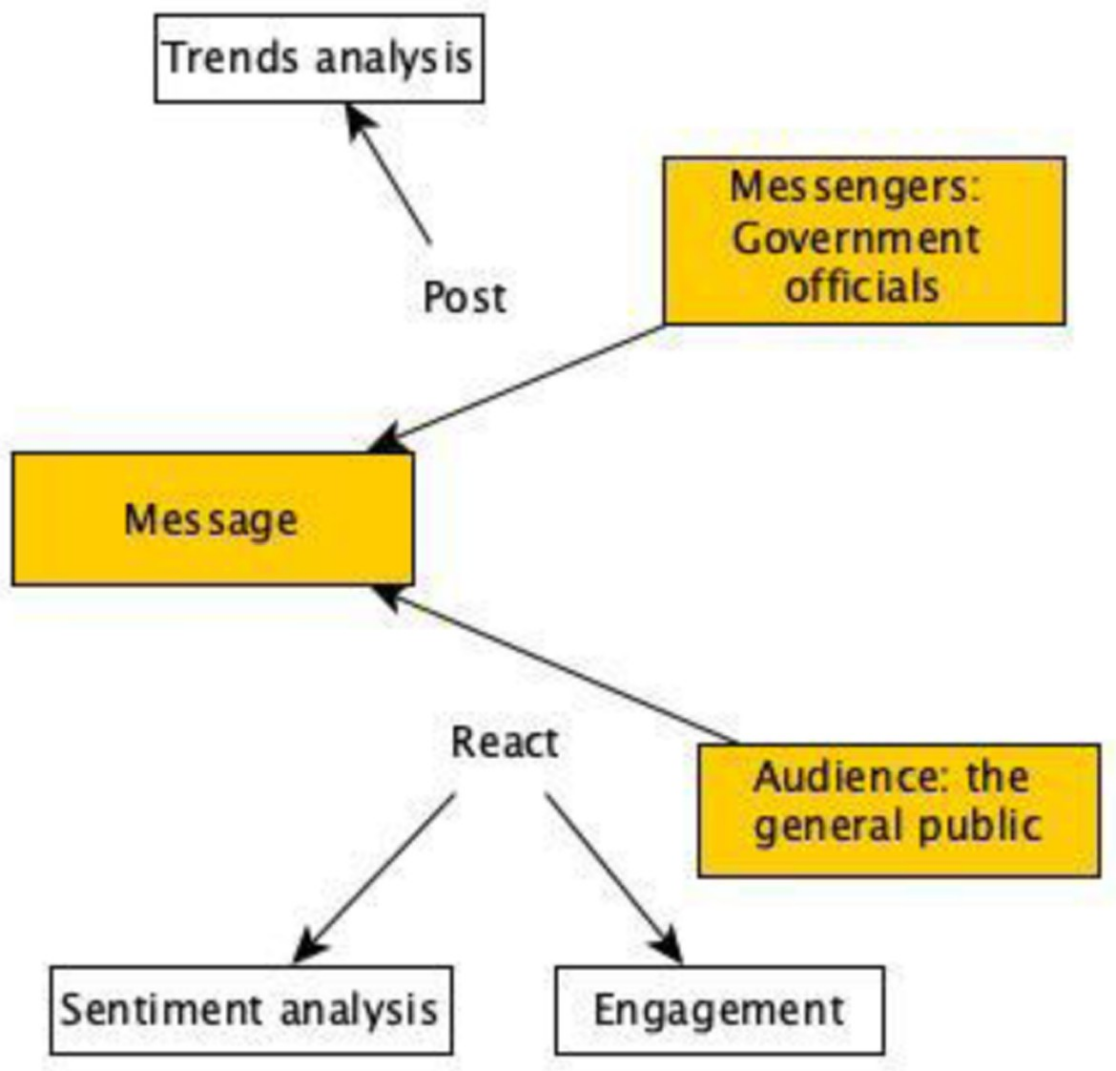
Conceptual model of the proposed research.

In the context of this research, we will look at the data posted by Canadian public officials addressed to the public and how the public reacted to Canadian public officials. In the first case, we will analyze the information communicated by governments to the public that we refer to as trends analysis. In the second case, we will look at the degree to which the public engaged with the messages posted by government officials (engagement) and how they reacted to them (sentiment). The conceptual model of this research is depicted in Fig 1.

### 3.3. Government and public health officials’ social media accounts

The messengers are the Canadian public officials that we canvassed in four categories of Twitter accounts: provincial/territorial and federal government officials’ accounts, respectively, and provincial/territorial and federal public health officials’ accounts, respectively. We selected these accounts since government and public health authorities led the dissemination of COVID-19-related information to the public in Canada.

For the provincial/territorial and federal government official accounts, we downloaded tweets from the official department/organizational accounts (e.g., @Canada, @ONgov, @GouvQc), as well as the individual accounts of the corresponding organizations’ leaders (e.g., Canada’s Prime Minister Justin Trudeau, Ontario’s Prime Minister Doug Ford, and Quebec’s Prime Minister François Legault). We replicated this model for the public health officials’ accounts, downloading tweets from organizational handles (e.g., @GovCanHealth, @ONThealth, @sante_qc) as well as the leaders of these organizations (e.g., Canada’s Minister of Health the Honourable Patty Hajdu, Ontario’s Minister of Health the Honourable Christine Elliot, and Quebec’s Minister of Health and Social Services Christian Dubé). In Appendix A in S1 File, we present in Tables 1, 2 in S1 File the full list of Twitter handles included to obtain study data.

To ensure the validity of selected accounts, we limited our data collection to Twitter-verified accounts [29]. Verified accounts are often destined for well-known organizations or individuals and are indicated by a blue verified badge that appears next to the account holder’s name. It is important to note that Twitter does not endorse posts from any account—verified or unverified.

### 3.4. Data access and format

The messages to be analyzed are tweets. Twitter offers two relevant Application Programming Interface (API) components to access tweets, data, and metadata. These applications are the Representational State Transfer (REST) API, used to retrieve past tweets matching established criteria within a search window available for Twitter searches; and the streaming API, used to subscribe to a continuous stream of new tweets matching the criteria, delivered via the API as soon as they become available. Each of these two APIs is offered by Twitter on three different levels, known as the standard API, the premium API, and the enterprise API [30]. To inform our study, we accessed tweets through the REST component of the premium API. We describe all the steps of data collection, filtering, and processing in Section 3.5.

Our data analysis was conducted using R, which is a programming language and free software for statistical computing and graphics commonly used by statisticians and data miners [31]. We used *rtweet*, which is a community-maintained R client for accessing Twitter’s REST and stream APIs [32] in order to access the data and metadata needed to perform our analysis.

Mining tweets through the Twitter REST Premium API provided us with the text of the tweets as well as with several metadata, including the sending user’s Twitter name and numerical ID, the time of posting, geolocation information (when available), and various data points which relate to the sender’s Twitter profile settings that we briefly describe in Appendix B in Table 3 in S1 File [30].

### 3.5. Data collection, filtering, and preprocessing

Between December 31, 2019, and August 31, 2020, we archived all the Twitter posts, replies, and associated metadata published by Twitter accounts presented in Appendix A in S1 File with no restriction. The dates of collected data are in the timeframe of the first wave of the COVID-19 pandemic in Canada, which was confirmed using Google Trends data [33, 34]. After the first step of data collection, we assessed 65,793 archived tweets and 80,256 archived replies.

As a second step, we filtered the collected tweets to retain those related to COVID-19. To this end, we filtered collected tweets based on the hashtags present in the tweets’ metadata. To facilitate this, we established a list of hashtags related to COVID-19 (see Appendix C in S1 File for the full list of included hashtags). To develop the list of COVID-19-related hashtags, we used relevant literature [35] and social media tools and guides [36–38]. Some of these hashtags were related specifically to the COVID-19 pandemic (e.g., #COVID-19, #2019nCov), others were related to public health messaging, to COVID-19 impacts (e.g., #StayHome, #StayHomeSaveLives), or related topics (e.g., #N95, #PPE which describe the required personal protective equipment needed during the pandemic). Included tweets were required to have at least one of these COVID-19 related topics to be retained in the dataset. Once the tweets were filtered, we collected all replies related to the retained tweets.

**Fig 2:**
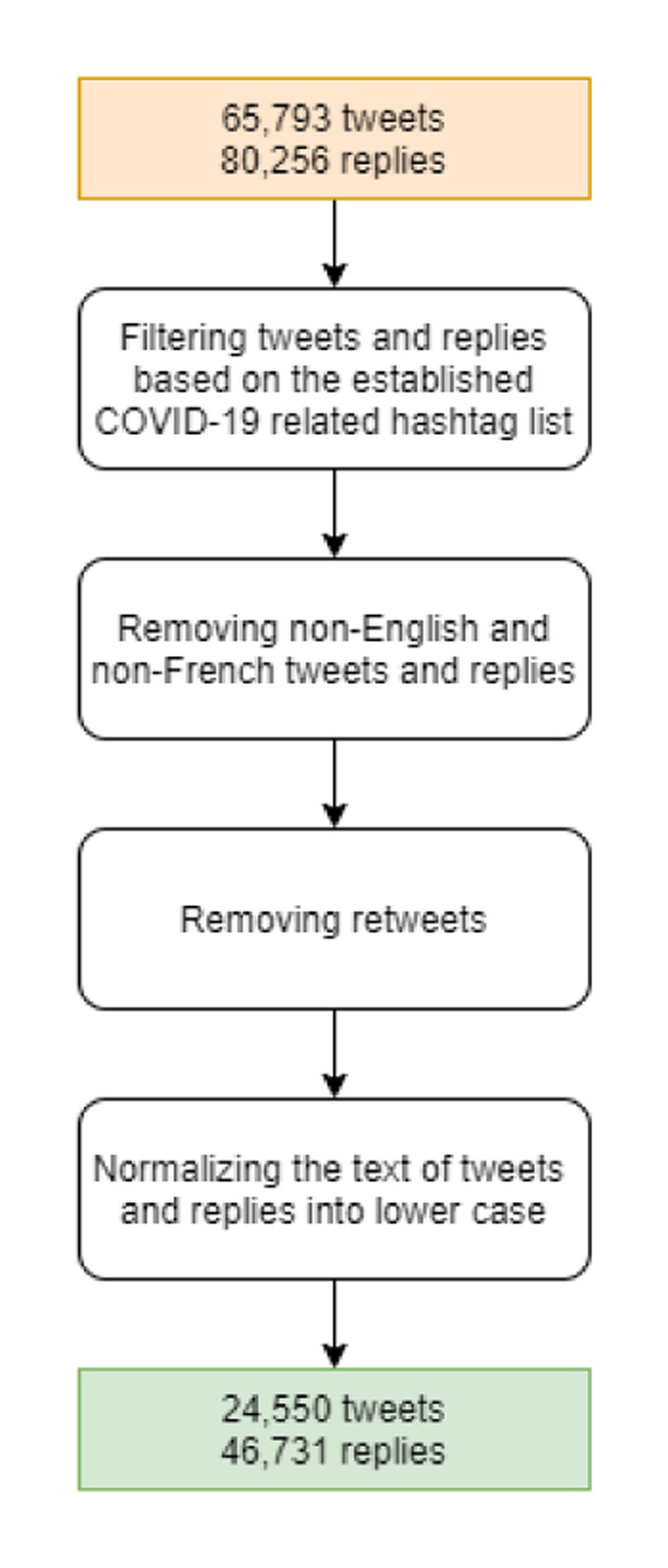
Data filtering and preprocessing workflow.

Finally, as a third step, we preprocessed the retained tweets and replies in order to use them in our data analysis. To this end, we first eliminated any non-English or non-French language tweets and replies from our dataset. Next, we removed retweets to reduce repetition in the dataset. Finally, we converted all tweets and reply text to lowercase to avoid duplication due to text case. At the end of these three steps, we have had a total of 24,550 tweets and 46,731 replies. The three-step workflow is depicted in Fig 2.

To give context to the analysis performed on the tweets and replies, we collected data related to the number of COVID-19 confirmed cases in Canada on a daily basis from December 31, 2019, to August 31, 2020. These data were obtained from the COVID-19 Data Repository maintained by the Center for Systems Science and Engineering (CSSE) at Johns Hopkins University [39].

We mention that in the context of this research, we were aware of the legal and ethical implications of collecting data from social media platforms. To this end, we first note that we used public data from Twitter. Second, we applied for permission from Twitter Inc. to have access to Twitter data. Finally, this study is designed based on anonymous feature account data rather than user-specific data. The data used was anonymous, and no personal data has been gathered or exploited for any purpose.

### 3.6. Data analysis

**Fig 3:**
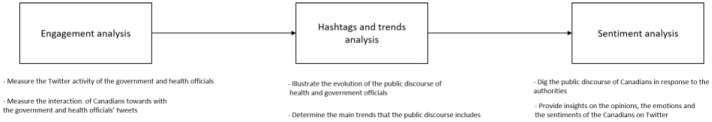
The data analysis process.

We performed three types of data analysis on our filtered dataset of COVID-19-related tweets (see Fig 3):

1. Analysis of the Canadian public’s engagement with government and public health officials’ tweets by looking at the number of retweets, the number of likes, and the ratio of interest.
2. Hashtag analysis to illustrate the evolution of the Canadian public discourse during the pandemic’s first wave.
3. Sentiment analysis to provide insights on the public’s reaction to the Canadian authorities’ tweets.

To compare reach of federal accounts (which target all people living in Canada) versus provincial/territorial accounts (which target people living in that region), we present aggregate findings across provincial/territorial government and public health accounts, respectively.

#### 3.6.1. Engagement metrics analysis

We established three engagement metrics with tweets: the number of retweets, the number of likes, and the ratio of interest. A retweet is a re-posting of a tweet; Twitter’s retweet feature helps users to quickly share a tweet with all their followers. Retweets can be considered as a sign of value as a user finds a tweet valuable enough to share it with their audience. Participants can also “like” Tweets by clicking on the heart icon. Likes can be considered as a sign of appreciation that users can express towards tweets. These metrics demonstrate the rate at which a tweet has been approved and shared by Twitter users. While the number of retweets and the number of likes are at the tweet level, the ratio of interest is at the user level. This ratio evaluates the interest to the tweets of a messenger. The ratio of interest is computed as the ratio of the number of interactions to the number of tweets, where the number of interactions is the sum of the number of likes and the number of retweets [40, 41]. The ratio of interest provides the average number of interactions for each tweet. To provide context to the data, we transposed these tweets against the evolution of COVID-19 confirmed daily cases in Canada during the study period.

#### 3.6.2. Hashtag trends analysis

A hashtag is used to index keywords or topics on Twitter. This function allows Twitter users to easily follow topics they are interested in. The hashtag trends analysis aims to show how the COVID-19 discourse evolved during the study period. The hashtag analysis was conducted by presenting the most popular hashtags in our dataset and by eliminating all the hashtags that were used to filter the tweets that we presented in Appendix C in S1 File. In doing so, we were able to observe the topic shift in this period. To contextualize the data, we crossed the evolution of trending hashtags over time with the number of daily COVID-19 confirmed cases in Canada.

#### 3.6.3. Sentiment analysis

The objective of sentiment analysis is to understand the emotions underlying a data source. Sentiment analysis is the computational and automated study of people’s opinions, sentiments, emotions, and attitudes towards products, services, issues, or events [42]. In this sense, sentiment analysis should allow understanding and tracking of the public’s “mood” about a particular entity to create actionable knowledge. This knowledge could be used to dig, explain, and predict social phenomena [43, 44]. Sentiment analysis can be conducted using an automated mining of attitudes, opinions, views, and emotions from text, speech, and database sources [45]. This mining is produced through Natural Language Processing (NLP) and Machine Learning (ML) techniques [46].

**Fig 4:**
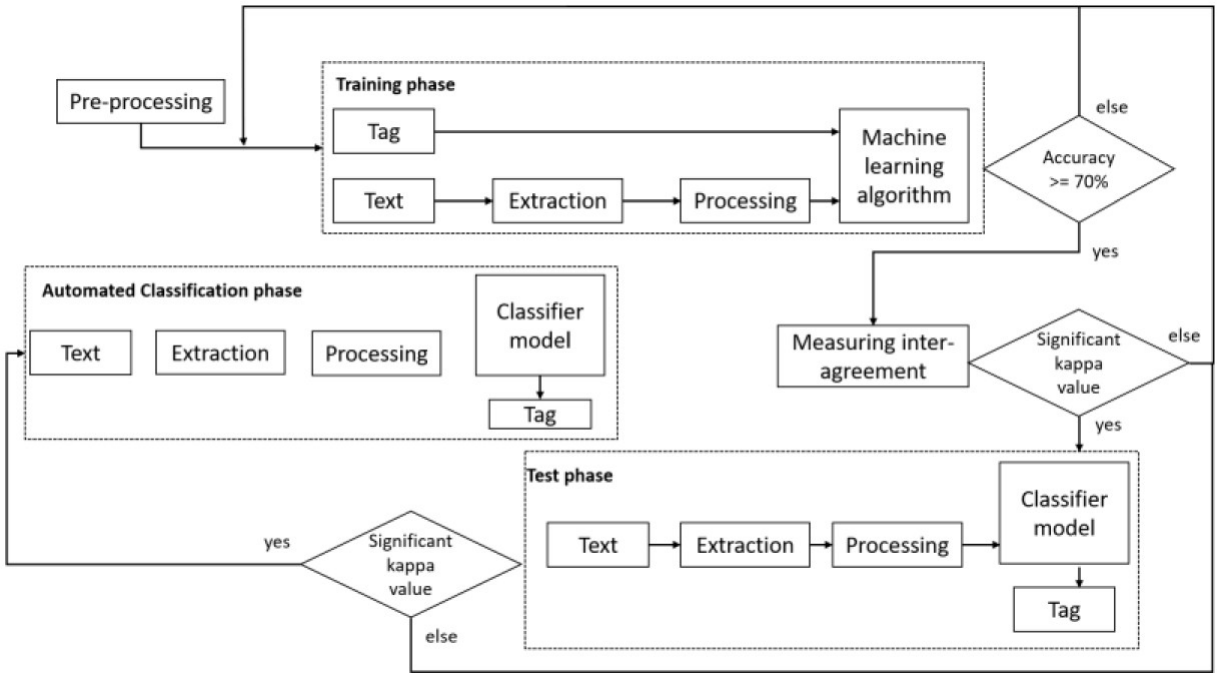
The overall sentiment analysis process.

In this study, we capture the public’s (the audience as a third component of risk communication) sentiments in response to government and public health officials’ tweets. The public response shows the importance of a two-way risk communication where we not only study Governments-Citizens communication channels but we also study the replies and reactions of the public to governments’ posts [8] We based our classification of tweet replies on the 10 sentiment categories identified in Chew & Eysenbach’s 2010 study of Twitter data during the H1N1 pandemic (See Appendix D in, Table 4 in S1 File) [47]. We adopted the sentiments identified by Chew & Eysenbach’s 2010 study since, to the best of our knowledge, it is the most complete classification of sentiments, and it was applied in a pandemic context which is the same as the context of this study. We refined these 10 sentiment categories to ensure that they captured the full spectrum of sentiments in our dataset. To do so, we first conducted a training phase, followed by an automated text classification phase. We used MonkeyLearn [48], an online machine learning platform, to perform this analysis (see Fig 4).

In the training phase, two researchers (AC, AJP) manually coded 1,424 randomly selected tweets replies from our existing dataset using the 10 sentiment categories. Where needed, the coders added new categories to capture new sentiments. The coders continued to conduct iterative rounds of coding until a Cohen’s Kappa of>0.6 was reached, which indicates a good level of coder agreement [49]. In the first round, using a dataset of 500 tweets from the 1,424 tweets, the Kappa coefficient was 0.46. The coders reconciled discrepancies and refined the coding framework using consensus, which included the addition or removal of sentiment codes. Using another set of 924 tweets, the coders independently categorized the sentiments and achieved a Cohen’s Kappa of 0.67 [50].

Following the training phase, we defined two new sentiment categories and adapted four categories from the Chew & Eysenbach framework. The two categories that we created are “racism and stigma” and “information sharing.” For the sentiment “racism and stigma,” we observed several tweets with racist and discrimination-based expressions. For the “information sharing” sentiment, we observed that users, in some replies, used to share news and information referring to the pandemic. In addition to these two new sentiments, we modified four categories from the Chew & Eysenbach framework as described hereafter:

- We changed the label of the category “misinformation” with “distrust.” We observed that the replies we initially categorized as misinformation were better represented as distrust (e.g., distrust of authority or the media).
- We changed the category labelled as “questions” with “information requests and inquiries,” as most of the questions that we observed in our data included help or clarification requests.
- We observed that replies of the category “personal opinion or interest” frequently expressed personal opinions along with suggestions (e.g., suggestions for COVID related policy changes or suggested public health measures). To capture both aspects, we revised the label of this category to “personal opinion or suggestion.”
- Initially, the category “resources” referred to replies pointing to additional information, beyond the tweet, such as a link to an article. However, in many instances, we found tweets providing additional information but without providing a link to an external resource. As a result, we revised this category to encompass both “information sharing and resources.”

The final 11 sentiment categories used in this paper, including definitions and examples, are listed in Table 1.

**Table 1:** The final categories of sentiments, their definition, and examples of tweets for each category.

Category	Definition	Examples
***Concern***	Replies that express COVID-19-related fear, anxiety, worry, or sadness for self or others. May also express skepticism.	“omg ppl stay home for the love of god” #stayathome
***Distrust***	Replies that contradict the reference standard or contain unsubstantiated information. May make speculations or express distrust of authority or the media. May include conspiracy theories or misinformation.	“Deflecting much? You lied about masks! It was all BS! You told Canadians we didn’t know how to safely wear mask” “#CoronaVirus #CoVid19 LancetGate: Big Pharma Corruption And Their COVID-19 Lies”
***Downplay***	Replies that attempt to de-emphasize the potential risks of COVID-19 or bring it into perspective. May also express a lack of concern or disinterest.	“there’s nothing to be afraid of.” “don’t forget to tell everyone that the normal flu has 2x the cases and 8 deaths this year”
***Frustration***	Replies that express anger, annoyance, scorn, or volatile contempt. May include coarse language.	“This team should be fired! Shame on you!!” “You are a disgrace and a fraud #RESIGN”
***Humour or sarcasm***	Comedic or sarcastic replies.	“You look so funny when you want to be credible” “you have a funny way of showing your appreciation”
***Information requests and inquiries***	Replies that include questions, demand clarifications or help.	“Here is a question: The man who died at his home of covid19 (not hospital), was he tested for covid 19?” “What measures exactly?”
***Information sharing and resources***	Replies containing COVID-19 news, updates, or any related information. May be a title or summary of a linked article.	“there are 598 cases in continuing care facilities, 921 cases at [location]”
***Personal experiences***	Replies where users mention a direct (personal) or indirect (e.g. family or acquaintance) experience with COVID-19.	“me and my wife are both feeling sick. sore throat. tired. minor cough. chest tightness. we work at [location]. so lots of exposure to the public. the phone line is busy.”
***Personal opinion or suggestion***	Replies where users express opinions about the COVID-19 pandemic (i.e., their perceptions of the SARS-CoV-2 virus, the COVID-19 situation or news) and provide suggestions.	“help the front-line staff and give them proper equipment including n95 masks. please communicate with the health minister” “while social distancing may be happening. self isolation isn’t. that is disappointing. please make sure people who are sick have the space they need to heal away from other people.”
***Racism and stigma***	Replies related to racist and discrimination-based expressions	“CCP restricted Wuhan ppl to go to Beijing in Jan 2020. Why? Because CCP knew that Wuhan Coronavirus was dangerous. CCP allowed Wuhan ppl to go to Canada, USA, etc in Jan 2020. Why? Because CCP used Wuhan Coronavirus as a bioweapon to attack the West. #ChinaLiedPeopleDied”
***Relief***	Replies that express joy, happiness, or sense of peace. May also express gratitude and acknowledgement.	“Please keep the great job that you are doing” “thank you! so glad you and your family are well. this is a reassuring message and so appreciated.”

Once we established our new sentiment analysis coding framework, we launched the trained machine learning classification model to analyze the remaining dataset. We first validated the machine algorithm using an agreement assessment and a machine learning classifier performance evaluation.

For the agreement validation, we compared manually coded tweets to machine-coded tweets. The Cohen’s Kappa coefficient value was 0.47; discrepancies were mainly attributed to the sentiments of “Concern” and “Frustration” which were not differentiated by the machine model. We enriched the training dataset with additional sentences for these categories and reran the analysis. Strong agreement between the manual and automated coding was achieved with a Cohen’s Kappa coefficient value of 0.74. Finally, for the machine learning validity evaluation, we measured the performance of the classifier using the following metrics:

- Accuracy: which is the number of correct predictions the classifier has made divided by the total number of predictions [51]. In our case, accuracy is 79%.
- Precision: which states the proportion of texts that were predicted correctly out of the ones that were predicted as belonging to a given category or tag [52]. Precision was computed at 65%.
- Recall: which is the ratio of texts that are correctly classified as belonging to a given category to the total number of texts of that category [52]. It shows the completeness of a given category with respect to each category [52]. The value of recall is 78%.
- F1 score: which is identified as a combination and harmonic means of precision and recall. This metric is widely adopted to evaluate the performance of the classification for each of the categories [52]. The greater the F1 score, the better the performance of our model. The F1 measure is 83%.

## 4. Results

### 4.1. Engagement metrics

**Fig 5:**
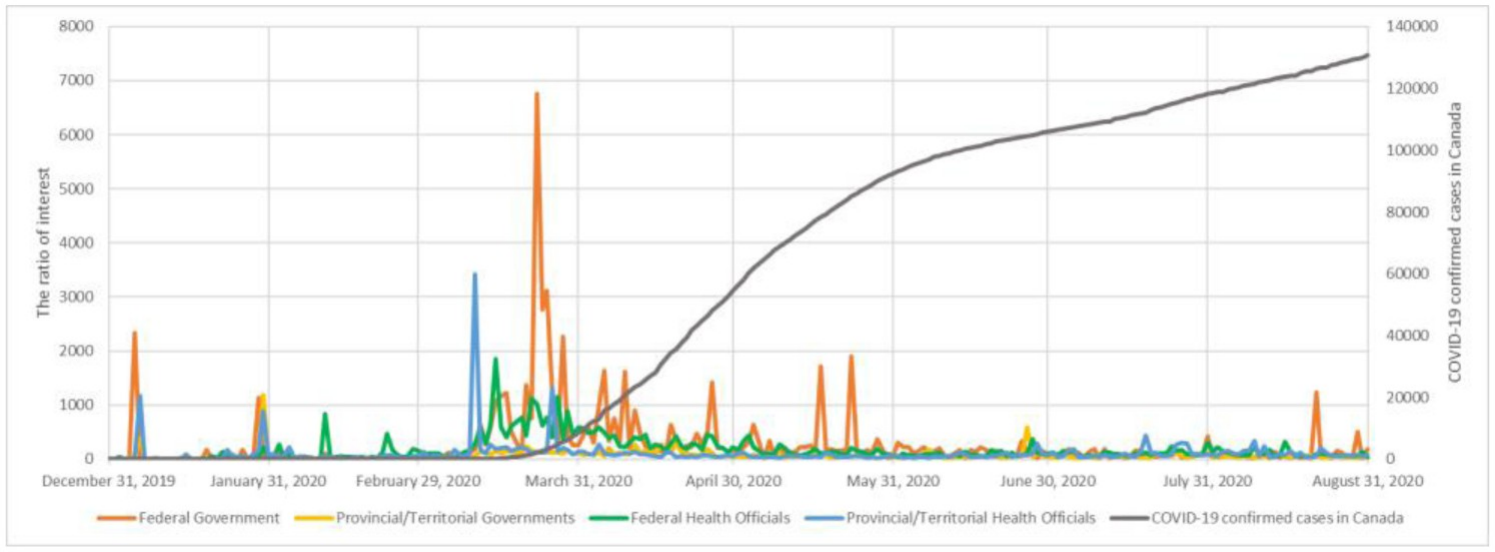
The ratio of interest [the ratio of the number of interactions to the number of tweets] towards the COVID-related tweets by Canadian officials x number of COVID-19 confirmed cases in Canada from December 31, 2019, to August 31, 2020.

In Fig 5 and in Table 2, we present the results related to the engagement metrics. In Appendix E in S1 File, we present in Figs 1–4 in S1 File the evolution of the number of tweets, likes, and retweets for the four different categories of accounts.

**Table 2:** Descriptive data showing the number of tweets, retweets, likes, and the ratio of interest related to COVID-related tweets by Canadian government and public health officials from December 31, 2019, to August 31, 2020.

Months	N. of cases in Canada	Federal Government Officials of Canada (3 Twitter accounts)				Government Officials of all Canadian Provinces and Territories (30 Twitter accounts)				Federal Health Officials of Canada (4 Twitter accounts)				Health Officials of all Canadian Provinces and Territories (56 Twitter accounts)			
		Tweets	Retweets	Likes	Ratio of interest	Tweets	Retweets	Likes	Ratio of interest	Tweets	Retweets	Likes	Ratio of interest	Tweets	Retweets	Likes	Ratio of interest
**Dec 2019**	0	0	0	0	-	0	0	0	-	0	0	0	-	0	0	0	-
**Jan 2020**	12	7	4,728	1,397	875	14	5,336	85	387.21	150	4,921	5,243	67.76	215	33,244	15,948	228.80
**Feb 2020**	234	10	537	187	72.40	52	829	43	16.77	218	17,939	5,969	109.67	367	12,713	3,559	44.34
**Mar 2020**	51,802	303	176,994	278,134	1,50207	1,492	97,721	90,486	126.14	812	193,767	275,694	578.15	2,835	646,234	155,052	282.64
**Apr 2020**	929,1	293	67,705	92,811	547.84	1,892	65,765	155,289	116.84	974	101,030	204,65	313.84	2,588	99,129	117,841	83.84
**May 2020**	2,368,790	202	20,474	29,214	245.98	1,435	25,064	48,562	51.31	1,115	49,036	103,091	136.44	1,920	40,353	56,395	50.39
**Jun 2020**	3,015,562	144	11,805	10,893	157.63	786	14,292	34,585	62.18	876	29,876	47,777	88.64	1,287	26,607	49,457	59.10
**Jul 2020**	3,460,185	77	5,161	2,252	96.27	664	12,722	27,338	60.33	659	25,685	45,952	108.71	1,119	32,453	72,028	93.37
**Aug 2020**	3,854,051	54	3,861	5,232	168.39	505	9,468	16,630	51.68	617	19,673	40,100	96.88	868	27,447	52,310	91.89

Provincial/territorial government officials of Canada tweeted a total of 6,840 COVID-related tweets from the 30 Twitter accounts analyzed (an average of 228 tweets per account during the study period). Federal government officials tweeted a total of 1,090 COVID-related tweets for a total of 3 Twitter accounts analyzed (an average of 363 tweets per account). The calculated ratio of interest of the federal government officials’ accounts was 652.65, which is higher than that of the provincial/territorial government officials of 88.34, meaning that the number of interactions per tweet were greater for federal government officials’ accounts compared to provincial/territorial governments’ accounts. This was observed for the entire duration of the study, with a peak of engagement with federal government officials’ accounts during the month of March.

Provincial/territorial health officials tweeted a total of 11,199 COVID-related tweets from 56 Twitter accounts analyzed (an average of 200 tweets per account). Federal health officials tweeted a total of 5,421 COVID-related tweets from 4 Twitter accounts analyzed (an average of 1355 tweets per account). The calculated ratio of interest of the federal health officials’ accounts was 215.90, which is higher than that of the provincial/territorial health officials at 128.65, meaning that the public in Canada engaged more with the federal health officials than with the provincial/territorial health officials. The periods that recorded the highest engagement with the federal health officials were the months of March and April. Furthermore, we observe that the ratio of interest for tweets generated by federal government officials was greater than the federal public health officials. However, we observe that the ratio of interest was higher for the provincial/territorial health official accounts as compared to provincial/territorial government officials.

Overall, the public demonstrated a greater level of engagement with federal Twitter accounts with an overall ratio of interest of 165.90 as compared to provincial/territorial Twitter accounts with an overall ratio of interest of 157.11. We also observe that, on average, each federal Twitter account tweeted more than a provincial/territorial Twitter account.

### 4.2. Hashtag trends

In Table 3, we present the results of the hashtag analysis, depicting the hashtag trends per month used by the Canadian officials. In Appendix F in S1 File, we present the top 10 hashtags for the four different categories of accounts.

**Table 3:** COVID-19 hashtag trends per month used by the Canadian government and health officials’ accounts from December 31, 2019, to August 31, 2020, demonstrating the topic shift.

Months	Dec 2019	*Jan 2020*	*Feb 2020*	*Mar 2020*	*Apr*. *2020*	*May 2020*	*Jun 2020*	*Jul 2020*	*Aug 2020*
**Hashtags and their occurrence**	N/A	MENTALHEALTH (44)	LONGTERMCARE (49)	FLATTENTHECURVE (238)	FLATTENTHECURVE (193)	PHYSICALDISTANCING (151)	PHYSICALDISTANCING (79)	TESTANDTRACE (60)	TESTANDTRACE (49)
		LONGTERMCARE (17)	MENTALHEALTH (20)	SOCIALDISTANCING (120)	PHYSICALDISTANCING (184)	MENTALHEALTH (96)	TESTANDTRACE (78)	MENTALHEALTH (44)	EPIDEMIOLOGY (44)
				PLANKTHECURVE (80)	STAYHOME (85)	TESTANDTRACE (67)	FLATTENTHECURVE (35)	PHYSICALDISTANCING (43)	MENTALHEALTH (37)
				PHYSICALDISTANCING (79)	PLANKTHECURVE (82)	FLATTENTHECURVE (65)	LONGTERMCARE (24)	EPIDEMIOLOGY (40)	PHYSICALDISTANCING (22)
				LONGTERMCARE (55)	MENTALHEALTH (65)	STRONGERTOGETHER (44)	MENTALHEALTH (22)	LONGTERMCARE (31)	LONGTERMCARE (17)
				SLOWTHESPREAD (51)	LONGTERMCARE (48)	PLANKTHECURVE (30)	EPIDEMIOLOGY (21)	DÉPISTAGE (17)	DÉPISTAGE (16)
				MENTALHEALTH (34)	PROTECTTHEVULNERABLE (43)	STAYHOME (25)	DÉPISTAGE (17)	PLANKTHECURVE (15)	STAYSAFE (6)
				STAYHOME (26)	TESTANDTRACE (42)	PROTECTTHEVULNERABLE (25)	PLANKTHECURVE (16)	FLATTENTHECURVE (10)	STRONGERTOGETHER (4)
				PROTECTTHEVULNERABLE (25)	TOGETHERAPART (42)	LONGTERMCARE (18)	TOGETHERAPART (15)	STRONGERTOGETHER (7)	STOPTHESPREAD (4)
				STRONGERTOGETHER (22)	STRONGERTOGETHER (36)	STOPTHESPREAD (16)	STRONGERTOGETHER (13)	TOGETHERAPART (4)	TOGETHERAPART (3)

Table 3 illustrates the topic shift over time in the COVID-related Canadian officials. We observe that, generally, the COVID-19-related Canadian discourse was consistent throughout the first wave of the pandemic, focusing on COVID-19 mitigation messages. We observe an immediate shift in public discourse from the period preceding the first wave of the COVID-19 pandemic (January and February) to the period of the first wave (beginning March 2020) with top trending tweets related to COVID-19 mitigation strategies (e.g., #SocialDistancing, #TestAndTrace and #StayHome) and COVID-19 mitigation goals (e.g., #FlattenTheCurve, #PlankTheCurve and #StopTheSpread); while during provincial lockdowns, we saw trends such as #StayAtHome emerge. Additionally, we observed changes in language over time; for instance, #SocialDistancing, which was trending in March 2020 was replaced by #PhysicalDistancing for the remainder of the study period. Throughout the period, we observed messages of solidarity and encouragement such as #StrongerTogether and #TogetherApart.

When looking at the trends data from each of the four account sources, we observed that provincial/territorial governments used more COVID-19-related hashtags compared to the federal government (443 versus 135 respectively). While all government accounts used hashtags related to COVID-19 mitigation strategies such as #PhysicalDistancing, we observed some trend differences. For instance, the federal government used hashtags on the economic fallout of the COVID-19 pandemic more often than provincial/territorial accounts (e.g., #EconomicResponse mentioned 21 versus 4 times by federal and provincial/territorial accounts, respectively). Additionally, provincial/territorial government accounts demonstrated greater use of hashtags related to mental health as compared to federal accounts (e.g., #MentalHealth mentioned 47 versus 10 times by provincial/territorial versus federal government accounts, respectively). Finally, we looked at the top 10 used hashtags during the period of data collection for the four types of accounts. We observed that federal public health officials used more often their top 10 COVID-19 hashtags compared to provincial/territorial public health officials (1,437 hashtags versus 1,280 hashtags, respectively). Similarly, all public health officials used hashtags related to COVID-19 mitigation strategies; however, there were slight differences. For instance, federal health officials highlighted the importance of testing and screening (e.g., #TestAndTrace and #Depistage) more often than provincial/territorial health authorities (374 versus 54 times, respectively).

### 4.3. Sentiment analysis

Table 4 presents the results related to the sentiment analysis of the overall collected tweets. In Appendix G in S1 File, we present in Figs 5–8 in S1 File the sentiment analysis for the four different categories of accounts.

**Table 4:** The proportions of expressed sentiments per month towards the government and health officials’ accounts from December 31, 2019, to August 31, 2020.

Sentiments	Concern	Distrust	Downplay	Frustration	Humour or sarcasm	Information request and inquiries	Information sharing and resources	Personal experiences	Personal opinion or suggestion	Racism and stigma	Relief
Months											
Dec 2019	0%	0%	0%	0%	0%	0%	0%	0%	0%	0%	0%
Jan 2020	0%	0%	0%	0%	0%	0%	0%	0%	0%	0%	0%
Feb 2020	16%	1%	1%	9%	1%	8%	4%	5%	6%	1%	4%
Mar 2020	24%	1%	2%	10%	2%	19%	14%	11%	14%	3%	12%
Apr 2020	21%	2%	3%	11%	1%	15%	12%	10%	12%	3%	12%
May 2020	20%	2%	4%	11%	1%	20%	11%	11%	12%	3%	17%
Jun 2020	23%	2%	2%	12%	2%	17%	13%	10%	11%	3%	11%
Jul 2020	23%	1%	3%	13%	1%	18%	11%	11%	13%	3%	13%
Aug 2020	25%	2%	2%	13%	1%	16%	14%	11%	14%	2%	14%
**Total sentiments**	**22%**	**1%**	**2%**	**11%**	**1%**	**16%**	**11%**	**10%**	**12%**	**2%**	**12%**

As stated in Section 3.6.3, we identified 11 sentiments in response to Canadian officials’ COVID-19-related tweets. The proportions of the overall sentiments were stable during the study period. The most commonly reported sentiment was *concern* at 22%, followed by *information requests and inquiries* at 16%. These were followed by *personal opinions or suggestions* (12%), *relief* (12%), *frustration* (11%), *information sharing and resources* (11%), and *personal experiences* (10%). Sentiments related to *downplay* and *stigma* were found in 2% of tweets, respectively, and sentiments related to *distrust* and *sarcasm* were found in 1% of tweets, respectively. When looking at these sentiments in federal versus provincial/territorial government accounts, we observe that the public expressed slightly more *concern* towards federal accounts (29% versus 23%, respectively). Users also demonstrated more *frustration* towards the federal government accounts (18% versus 12%). In contrast, sentiments for *information sharing and resources* were greater in response to provincial/territorial government accounts compared to the federal government (11% versus 6%, respectively). On the other hand, we observe that Twitter users expressed slightly more sentiments of *concern* towards provincial/territorial health officials (24%) compared to federal health officials (18%). Additionally, the public posted more *information requests and inquiries* in response to federal health officials’ tweets (20%) compared to provincial/territorial health officials (14%). Finally, when comparing federal accounts, we observed that the public expressed more *concern* and *frustration* towards federal government officials (29% and 18%, respectively) compared to federal public health officials (18% and 9%, respectively). The positive sentiment of *relief* was expressed slightly more for federal health officials (14%) compared to provincial/territorial health officials (10%). This sentiment was comparable for provincial/territorial government and public health officials (12% and 13%). Rates of *concern* expressed in response to all provincial/territorial accounts were comparable (23% and 24%), representing one quarter of all sentiments. We present in Appendix G in S1 File the detailed results that depict the evolution of sentiments over time.

## 5. Discussion

### 5.1 General observations

Our results about the hashtag trends analysis showed that COVID-19 discourse in Canada generally remained stable during the first wave of the pandemic. We observed subtle differences between government and provincial/territorial accounts, mostly related to frequency of use of certain hashtags (e.g., provincial/territorial governments placed greater emphasis on #MentalHealth messaging as compared to federal officials). We also observed subtle changes in language over time; for instance, the hashtag of #SocialDistancing was quickly replaced with #PhysicalDistancing, which was sustained for the duration of the study period.

Regarding the sentiment analysis, we made three main observations. First, we identified that distrust is 1% to 2% of the sentiment analysis. This ratio is quite low compared to emergent data from countries such as the United Kingdom where surveys showed 31% of the population did not trust the government to control the spread of the pandemic [53]. Balaet et al. further showed that 22% of study participants believed there were “ulterior motives” behind the government’s COVID-19 response, with minority populations also tending to show distrust towards the government [54]. For instance, our results are well aligned with a larger study that took place on Twitter, showing that Canada is one of the most trusted countries by its citizens in terms of COVID’19 management (score of 4.1 with 5 representing the highest scoring metric) [55]. Further understanding the factors that drive trust or distrust towards governments during public health emergencies can strengthen response efforts going forward. Second, we observed that approximately 2% of all sentiments were related to racism or stigma. Governments and public health leaders should embed an equity lens in the development of their key messages to discredit such sentiments and reduce discrimination. For instance, we observed trending of the hashtag #ProtectTheVulnerable. Despite positive intentions, potentially stigmatizing terms such as “vulnerable” should be avoided in public discourse, particularly by government and health official accounts (http://www.bccdc.ca/Health-Info-Site/Documents/Language-guide.pdf). White papers such as the Chief Public Health Officer of Canada’s Report on the State of Public *Health in Canada 2020* highlight the importance of embedding equity into policies and language to dismantle systems of oppression and racism in Canada, which were exacerbated during the pandemic [56–58]. Additional guidance such as the British Columbia Centre for Disease Control provides guidelines on using inclusive language for written and digital content [59, 60]. Approaching public health messaging using an equity and inclusivity lens may foster trust, particularly for historically marginalized populations and groups. For example, and as stated in the hashtag trends, we observed some changes in discourse over the course of the pandemic, for instance with the shift from #SocialDistancing to #PhysicalDistancing. Finally, we noted the public’s appetite for clear guidance on how to navigate the pandemic. Notably, approximately 33% of all sentiments were related to information requests or sharing. Leaders can leverage the public’s desire for such information by providing specific, plain-language, evidence-based recommendations to the public in real time. Specifically, the federal government and federal health accounts should disseminate this information, given the levels of public engagement that we observed towards these accounts. In this context, and to increase the visibility of their messages, government officials should leverage hashtags to better communicate with people. Studies suggest that leveraging “organically developed” hashtags, rather than creating new ones, may improve the visibility of certain messages [61]. Besides hashtags, government officials can engage influencers and experts in online conversations on social media [61] to help share verified facts about COVID-19 while aiming to reduce fear and anxiety.

### 5.2 Contributions to the literature

This research has four main contributions to the literature. First, our findings showed that the public demonstrated a greater ratio of interest towards the federal officials’ accounts compared to provincial/territorial officials’ accounts; this was observed for both government and health officials’ accounts. We hypothesized that the public would be more trusting of higher levels of government. Our results are consistent with the Statistics Canada crowdsourcing surveys which showed that, during COVID-19, Canadians were more trusting of the federal government compared to lower levels (61.5%, 55.8%, 54.7% trust in federal, provincial/territorial, and municipal governments, respectively). Also, Canadians were more trusting of health authorities compared to government (74.4%, 74.3%, 65.1% trust in federal, provincial/territorial, and municipal health authorities, respectively) [62]. Thus, as the perceived most trustworthy source by Canadians, federal health authorities should be equipped with timely, relevant, and actionable messaging during health emergencies.

Second, we noted that public health messaging between government and health authorities was not always consistent. For instance, provincial/territorial government accounts focused more on mental health compared to federal governments. Additionally, federal public health authorities used more hashtags related to testing and screening compared to provincial/territorial health authorities. While the differences we observed were minor, the findings provide an opportunity for reflection on how public trust is impacted by conflicting or inconsistent messaging. Risk messaging that follows established evidence-based guidance, such as the World Health Organization’s COVID-19 outbreak communication guidelines [63], may mitigate opportunities for misinformation while fostering public trust. Such recommendations include establishing trust with communities ahead of health emergencies, establishing relationships with partners (e.g., varying levels of governments or health authorities) to facilitate the rapid development and announcement of public health guidance, and planning to identify spokespersons and lead agencies (e.g., federal health authorities) to gain buy-in with politicians and other stakeholders.

Our study also explored public engagement with health and government authorities through a triangulation of varying data sources and methods, specifically an engagement analysis, hashtags and trends analyses, and a sentiment analysis. To the best of our knowledge, there are limited examples in the literature that used multiple sources as we have presented here [64, 65]. Rather, identified studies focused on a singular form of analysis, typically engagement, or sentiment analysis only [66–69]. Combining our sources provided additional depth to our research findings and demonstrated the feasibility of using machine learning methods to inform public health responses. Future work can continue to build on the methods we’ve presented here. For instance, initiatives such as the Early AI-supported Response with Social Listening study tracks real time COVID-19 related discussions in over 30 countries (https://www.who-ears.com/#/). Additional research to explore public sentiments by population groups [70, 71] (e.g., gender, age groups, newcomer groups) can provide insights on how to optimize messaging targeting such groups.

Finally, our study adds a methodological contribution in advancing the sentiment categories developed by Chew & Eysenbach (2010) to analyze Twitter discourse during the H1N1 outbreak. Specifically, we added two new categories and updated four existing categories. In contrast to the authors’ analysis approach of an automated sentiment classification based on search queries of keywords and phrases, we applied the Support Vector Machine algorithm which is the dominant algorithm for sentiment analysis [43]. We demonstrate therefore the feasibility of using natural language programming and machine learning techniques to analyze large social media datasets. Importantly, these techniques highly correlated with manually coded sentiments, demonstrating the validity of the approach. Such methods can be leveraged by government, health officials, and researchers to gauge real-time public sentiments during public health emergencies and craft corresponding messages.

### 5.3 Limitations

Our study is not without limitations. First, our findings are limited to the public discourse over Twitter, which may not be reflective of the overall Canadian public discourse across communication channels. Second, we restricted our study to English and French Twitter posts only, which may limit the representativeness of our results given the diversity of the Canadian population. Third, we are aware that there may be a selection bias in the collection of our data set as we only considered gathering tweets and responses posted by Canadian government and health officials only, thus overlooking other Twitter accounts that may have high traction and influence in the Twittersphere (e.g., celebrities, influencers, community leaders, religious leaders). Additionally, our data set was limited to tweets and replies that contained specific COVID-related hashtags only, which we developed using relevant literature [35] and social media tools and guides [36–38]. There is a possibility that we may have overlooked additional hashtags that may have been relevant to this discourse. Fourth, the interpretation of both hashtags and emojis usages during the classification phase of our sentiment analysis is limited by our lack of knowledge of how the authors intended them. Both are influenced by the context, the culture, the age and the gender of the authors, therefore making them open to interpretation [72, 73]. We observed a number of available emojis where each of them expresses a particular sentiment and that are more and more used. So, the combination of emojis with hashtags and sentiments can provide new substantial information to identify the sentiments behind a post. Consequently, it will be interesting to develop techniques to better interpret hashtags and emojis based on their context. Fifth, we did not disaggregate data by province/territory; additional research to do so would provide us with more insight on regional and context-specific considerations. Finally, our trends analysis only looked at the messages posted by governments in a one-way communication. Future research can investigate two-way communication models between the public and health/government authorities. Such two-way communication is critical to optimizing risk communications [8]. These limitations do not diminish the originality and the impact of our research, which resulted in the development of practical recommendations and important insights to support leaders to communicate in future crises.

## 6. Conclusion

We demonstrated the feasibility of leveraging machine learning and natural language programming to assess public discourse during a public health emergency using social media. Our findings suggest members of the Canadian public demonstrated increased engagement with federal officials’ Twitter accounts as compared to provincial/territorial accounts. Hashtag trends analyses illustrated the topic shift in the Canadian public discourse, which initially focused on COVID-19 mitigation strategies and evolved to address emerging issues such as COVID-19 mental health effects. Additionally, we identified 11 sentiments in response to officials’ COVID-19-related posts. We provided suggestions on how government and public health officials can optimize their messaging during public health emergencies. Future research can explore these trends in the context of the second and third waves, to determine the discourse of officials and the public over time in Canada.

## Supporting information

S1 File (DOCX)
