# Supplementary material for: An exploration of Canadian government officials’ COVID-19 messages and the public’s reaction using social media data
Source: PLoS One. 2022 Sep 2;17(9):e0273153. doi: 10.1371/journal.pone.0273153 (PMC9439209; doi:10.1371/journal.pone.0273153)
Supplement: S1 File — (DOCX) [file pone.0273153.s001.docx]

- 1. **Appendix A**

List of all List of all Twitter handles included to obtain data to inform the study.

| **Region/Country** | **Official Government Account** | | **Government Leader** | |  |
| --- | --- | --- | --- | --- | --- |
|  | **Account Name** | **Account Handle** | **Name/Account Name** | **Account Handle** |  |
| **Canada** | Canada | @Canada | Justin Trudeau | @JustinTrudeau |  |
|  |  |  | CanadianPM | @CanadianPM |  |
|  |  |  |  |  |  |
| **British Columbia** | BC Government News | @BCGovNews | John Horgan | @jjhorgan |  |
|  |  |  |  |  |  |
|  |  |  |  |  |  |
|  |  |  |  |  |  |
|  |  |  |  |  |  |
|  |  |  |  |  |  |
| **Alberta** | Alberta Government | @YourAlberta | Jason Kenney | @jkenney |  |
|  |  |  |  |  |  |
|  |  |  |  |  |  |
|  |  |  |  |  |  |
| **Saskatchewan** | Government of Saskatchewan | @SKGov | Scott Moe | @PremierScottMoe |  |
|  |  |  |  |  |  |
|  |  |  |  |  |  |
| **Manitoba** | Manitoba Government | @MBGov | Brian Pallister | @BrianPallister |  |
|  | Manitoba Government News | @MBGovNews |  |  |  |
| **Ontario** | Ontario Government | @ONGov | Doug Ford | @fordnation |  |
|  |  |  |  |  |  |
|  |  |  |  |  |  |
|  |  |  |  |  |  |
|  |  |  |  |  |  |
|  |  |  |  |  |  |
|  |  | @ONgouv |  |  |  |
|  |  |  |  |  |  |
|  |  |  |  |  |  |
|  |  |  |  |  |  |
| **Quebec** | Government du Quebec | @GouvQc | Francois Legault | @francoislegault |  |
|  |  |  |  |  |  |
| **Nova Scotia** | Nova Scotia Gov. | @nsgov | Stephen McNeil | @StephenMcNeil |  |
|  |  |  |  |  |  |
|  |  |  |  |  |  |
| **New Brunswick** | Government of New Brunswick | @Gov_NB | Blaine Higgs | @BlaineHiggs |  |
|  |  |  |  |  |  |
|  |  | @Gouv_NB |  |  |  |
|  |  |  |  |  |  |
|  |  |  |  |  |  |
| **Prince Edward Island** | Government of PEI | @InfoPEI | Dennis King | @dennyking |  |
|  |  |  |  |  |  |
| **Newfoundland & Labrador** | Government of NL | @GovNL | Dwight Ball | @DwightBallNL |  |
|  |  |  | Premier of NL | @PremierofNL |  |
|  |  |  |  |  |  |
|  |  |  |  |  |  |
| **Yukon** | Government of Yukon | @yukongov | Sandy Silver | @Premier_Silver |  |
|  |  | @LeYukonFr |  |  |  |
| **Northwest Territories** | Northwest Territories Government | N/A | Caroline Cochrane | @CCochrane_NWT |  |
|  |  |  |  |  |  |
| **Nunavut** | Government of Nunavut | @GOVofNUNAVUT | Joe Savikataaq | @JSavikataaq |  |
|  |  |  |  |  |  |
|  |  |  |  |  |  |

Table 5: List of all Twitter handles related to the official government Twitter accounts and government leaders Twitter accounts included to obtain data to inform the study.

| **Region/Country** | **National/Provincial Health Body** | | **National/Provincial Health Body Leader** | | |
| --- | --- | --- | --- | --- | --- |
|  | **Name** | **Account Handle** | **Name/Account Name** | **Title** | **Account Handle** |
| **Canada** | Health Canada | @GovCanHealth | Patty Hajdu (Health Canada) | Canada_Minister of Health | @PattyHajdu |
|  | Public Health Agency of Canada |  | Dr. Theresa Tam (PHAC) | Canada_Chief Public Health Officer | @CPHO_Canada |
|  | Santé Canada | @GouvCanSante |  |  |  |
| **British Columbia** | BC Centers for Disease Control | @CDCofBC | Dr. Bonnie Henry | BC_Provincial Health Officer | N/A |
|  | Ministry of Health | N/A | Adrian Dix | BC Minister of Health | @adriandix |
|  | Ministry of Mental Health and Addictions | N/A | Judy Darcy | BC_Minister of Mental Health and Addictions | @DarcyJudy |
|  | Ministry of Children and Family Development | N/A | Katrine Conroy | BC_Minister for Children and Family Development | @KatrineConroy |
|  | BC_First Nations Health Authority | @fnha |  |  |  |
|  | Provinical Health Services Authority of BC | @PHSAofBC |  |  |  |
| **Alberta** | Alberta Health Services | @AHS_media | Dr. Deena Hindshaw | Alberta_Chief Medical Officer of Health Alberta | @CMOH_Alberta |
|  | Alberta Health | @GoAHealth | Tyler Shandro | Alberta Minister of Health | @shandro |
|  | Ministry of Children's Services | N/A | Rebecca Shulz | Alberta_Minister of Children's Services | @rebeccakschulz |
|  | Alberta_Ministry of Community and Social Services | @AlbertaCSS | Rajan Sawhney | Alberta_Minister of Community and Social Services | @RajanJSaw |
| **Saskatchewan** | Saskatchewan_Ministry of Health | @SaskHealth | Jim Reiter | Minister of Health | N/A |
|  | Saskatchewan_Ministry of Rural and Remote Health | N/A | Warren Kaeding | Minister of Rural and Remote Health | N/A |
|  | Saskatchewan Health Authority | @SaskHealth | Dr. Saqib Shahab | Chief Medical Officer of Health Saskatchewan | N/A |
| **Manitoba** | Ministry of Health, Seniors and Active Living | N/A | Cameron Friesen | Manitoba_Minister of Health, Seniors and Active Living | @CameronFriesen |
|  |  |  | Dr. Brent Roussin | Manitoba_Chief Provinical Public Health Officer | @roussin_brent |
| **Ontario** | Ontario Ministry of Health | @ONTHealth | Christine Elliot | Ontario_Minister of Health | @celliottability |
|  | Santé Ontario | @ONTSante |  |  |  |
|  | Ontario Ministry of Long-Term Care | @Onlongtermcare | Merrilee Fullerton | Ontario_Minister of Long-Term Care | @DrFullertonMPP |
|  | Ontario Soin | @soinsldureeON |  |  |  |
|  | Public Health Ontario | @PublicHealthON | Dr. David Williams | Chief Medical Officer of health | N/A |
|  | Ontario_Ministry of Education | @ONeducation | Stephen Lecce | Ontario_Minister of Education | @Sflecce |
|  | Ontario_children | @ChildrenON | Todd Smith | Ontario_Minister of Children, Community and Social Services | @ToddSmithPC |
|  | Ontario_enfants | @enfantsON |  |  |  |
|  | Ontario_social_care | @ONSocialService |  |  |  |
|  | Ontario_aide_sociale | @ONaidesociale |  |  |  |
| **Quebec** | QC_Ministry of Health and Social Services | @sante_qc | Danielle McCann | QC_Minister of Health and Social Services | @MinistreMcCann |
|  | L'Institut national de santé publique du Québec | @INSPQ | Dr. Horacio Arruda | QC_National Director of Public Health and Assistant Deputy Minister of Public Health | @ArrudaHoracio |
| **Nova Scotia** | NS_Ministry of Health and Wellness | @nshealth | Randy Delorey | NS_Minister of Health and Wellness | @RandyDelorey |
|  | Nova Scotia Health Authority | @HealthNS | Dr. Robert Strang | NS_Chief Medical Officer of Health | @StrangRobert |
|  | NS_Ministry of Environment | @ns_environment | Gordon Wilson | Minister of the Environment | N/A |
| **New Brunswick** | NB_Department of Health | @NBHealth | Ted Flemming | NB_Minister of Health | @tedflemming |
|  |  | @SanteNB | Jennifer Wylie-Russell | Chief Medical Officer of Health | N/A |
|  | Department of Social Development | N/A | Dorothy Shephard | NB_Minister of Social Development | @ShephardDorothy |
|  | Department of Justice and Public Safety | N/A | Andrea AndersonMason | NB_Minister of Justice | @Onemainstreet |
|  | Department of Environment and Local Government | N/A | Jeff Carr | Minister of Environment and Local Government | N/A |
| **Prince Edward Island** | Department of Health and Wellness | N/A | James Aylward | PEI_Minister of Health and Wellness | @jsjaylward |
|  | Health PEI | @Health_PEI | Dr. Heather Morrison | Chief Public Health Officer | N/A |
| **Newfoundland & Labrador** | NL_Department of Health and Community Services | @HCS_GovNL | John Haggie | NL_Minister of Health and Community Services | @Johnrockdoc |
|  | NL_Dpartment of Children, Seniors and Social Development | @CSSD_GovNL | Lisa Dempster | NL_Minister of Children, Seniors and Social Development | @LisaVDempster |
|  | Service NL | @ServiceNL_ | Sherry Gambin-Walsh | Minister of Service NL | @Sgambin |
|  |  |  | Dr. Janice Fitzgerald | Chief Medical Officer of Health | N/A |
| **Yukon** | Yukon_Department of Health and Social Services | @HSSYukon | Pauline Frost | Yukon_Minster for Health and Social Services | @PFrostOldCrow |
|  |  |  | Dr. Brendan Hanely | Chief Medical Officer of Health | N/A |
| **Northwest Territories** | Department of Health and Social Services | N/A | Diane Thom | Minister of Health and Social Services | N/A |
|  | Northwest Territories Health and Social Services Authority | N/A | Kami Kandola | NWT_Chief Public Health Officer | @NWT_CPHO |
| **Nunavut** | Department of Health | N/A | George Hickes | Minister of Health | N/A |
|  |  |  | Dr. Michael Patterson | Chief Medical Officer of Health | N/A |

Table 6: List of all Twitter handles related to the health bodies Twitter accounts and healt body leaders Twitter accounts included to obtain data to inform the study.

- 1. **Appendix B**

Description of some *metadata* of a tweet.

Table 7: Description of some metadata of a Twitter post.

- 1. **Appendix C**

List of hashtags related to COVID-19 used to filter the tweets.

#wuhanvirus #CDC #Wuhan #Outbreak #ChinaKoronavirus #N95 #KungFlu #Epidemic #Sinophobia #Pandemic #Coronapocalypse #CancelEverything #Coronials #Panic #SocialDistancingbuying #DuringMy14DayQuarantine #Panicshopping #InQuarantineSurvivalKit #chinesevirus #stayhomechallenge #DontBeASpreader #shelteringinplace #trumppandemic #PPEshortage #saferathome #GetMePPE #covidiot #epitwitter #Pandemie #quarantineandchill #ImDoingFineBecause #safehands #PandemicIn5Words #see10send10 #seeapupseandsendapup #firstpicchallenge #QuarantineandChill #LockdownNow #Covidiots #MyPandemicSurvivalPlan #TogetherAtHome #BigOnlinePar #StaySafeStayHome #INoLongerRemember #Lockdown #COVIDIOTS #WFH #WorkingFromHome #COVID-19 #covid19 #2019nCov

- 1. **Appendix D**

The initial categories of sentiments, their definition, and examples of tweets for each category.


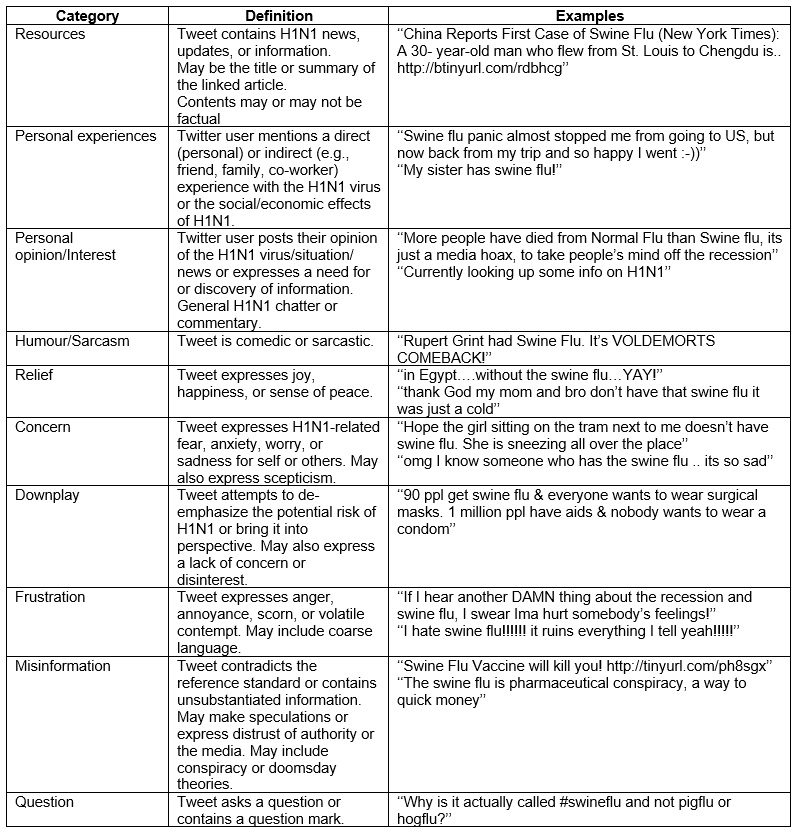


Table 8: The initial categories of sentiments, their definition, and examples of tweets for each category.

- 1. **Appendix E**

Engagement metrics results towards the COVID-related Canadian public discourse.


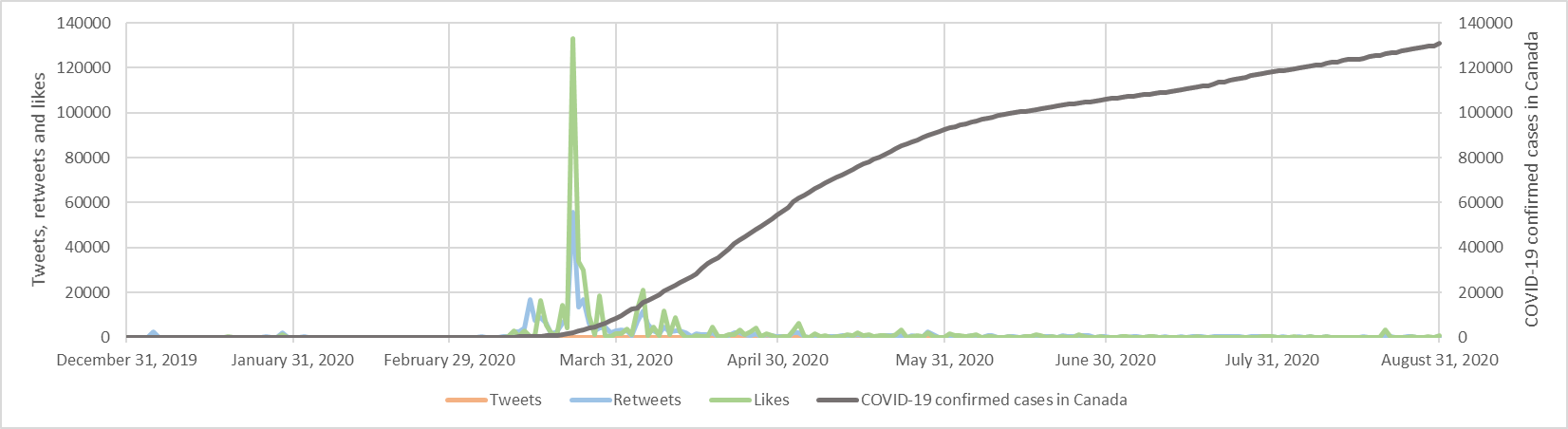


Figure 6: Tweets, retweets, and likes of COVID-related tweets by Canadian Federal government officials x number of COVID-19 confirmed cases in Canada from December 31, 2019, to August 31, 2020.


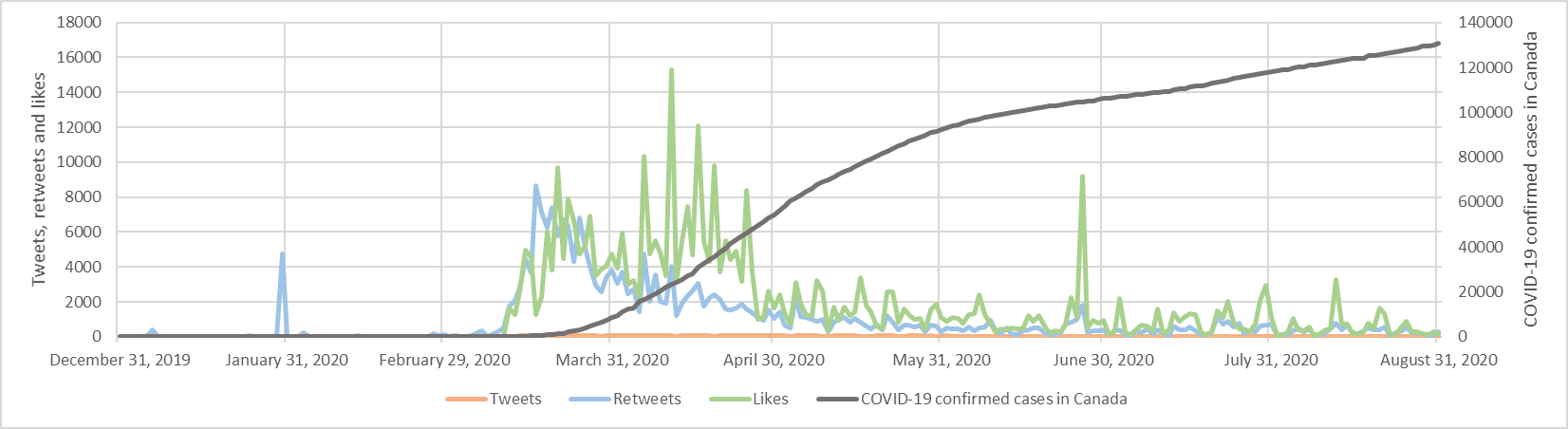


Figure 7: Tweets, retweets, and likes of COVID-related tweets of Canadian provincial/territorial government officials x number of COVID-19 confirmed cases in Canada December 31, 2019, to August 31, 2020.


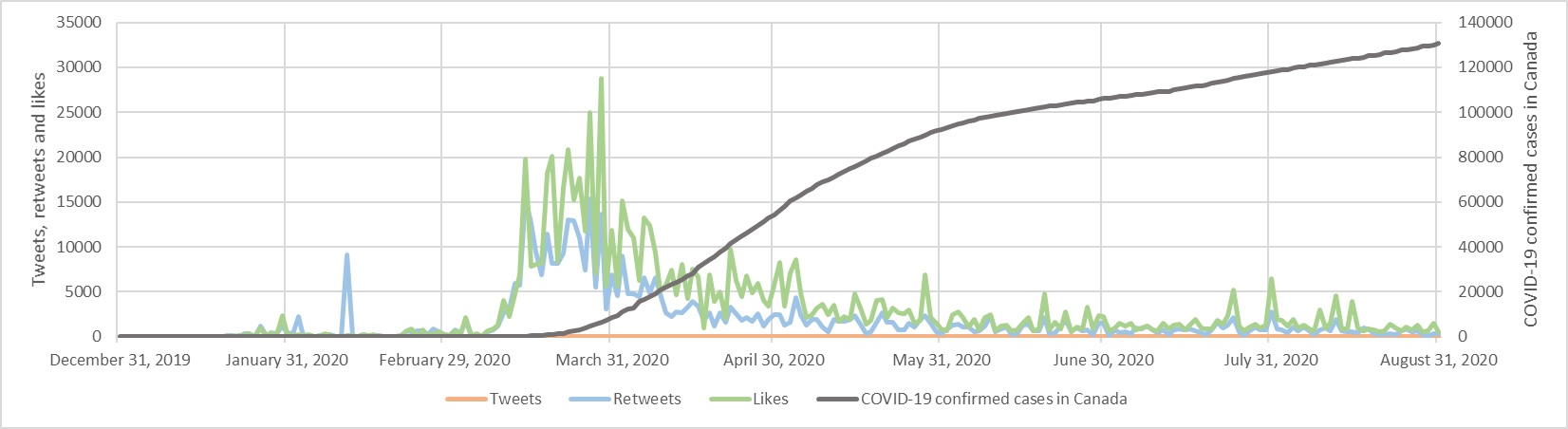


Figure 8: Tweets, retweets, and likes of COVID-related tweets of Canadian Federal public health officials x number of COVID-19 confirmed cases in Canada December 31, 2019, to August 31, 2020.


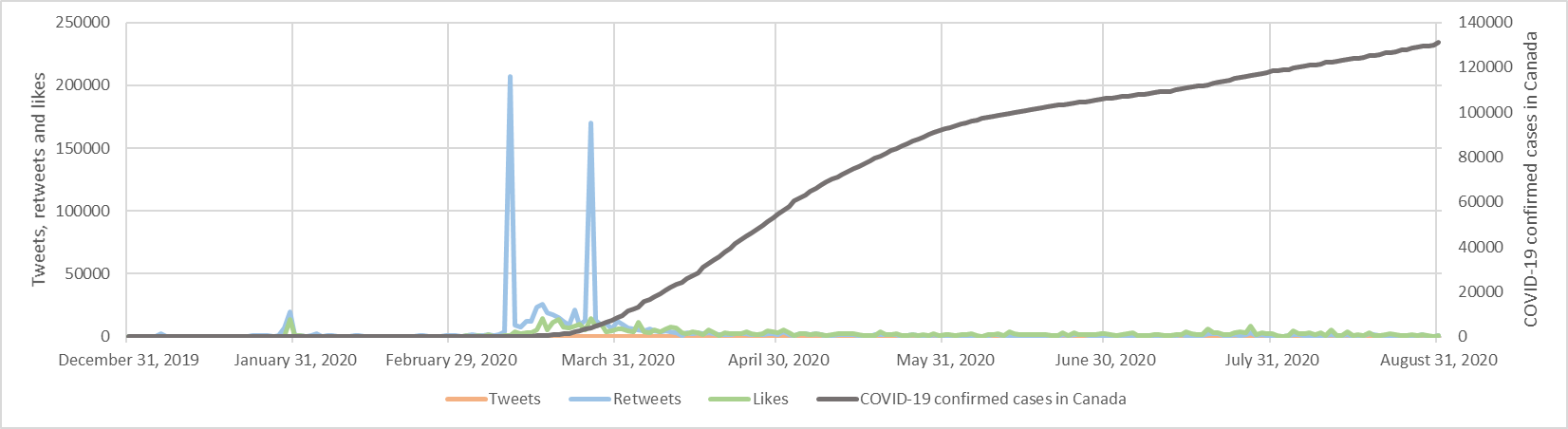


Figure 9: Tweets, retweets, and likes of COVID-related tweets of Canadian provincial/territorial public health officials x number of COVID-19 confirmed cases in Canada December 31, 2019, to August 31, 2020.

- 1. **Appendix F**

| **Accounts** | **Top 10 hashtags (by account)** |
| --- | --- |
| Provincial/territorial public health officials | LONGTERMCARE |
|  | MENTALHEALTH |
|  | FLATTENTHECURVE |
|  | SOCIALDISTANCING |
|  | PHYSICALDISTANCING |
|  | STAYHOME |
|  | STRONGERTOGETHER |
|  | PLANKHECURVE |
|  | STOPTHESPREAD |
|  | WASHYOURHANDS |
| Federal public health officials | TESTANDTRACE |
|  | PHYSICALDISTANCING |
|  | PLANKHECURVE |
|  | EPIDEMIOLOGY |
|  | PROTECTTHEVULNERABLE |
|  | TOGETHERAPART |
|  | STRONGERTOGETHER |
|  | SLOWTHESPREAD |
|  | FLATTENTHECURVE |
|  | DÉPISTAGE |
| Provincial/territorial government officials | FLATTENTHECURVE |
|  | STAYHOME |
|  | STAYSAFE |
|  | MENTALHEALTH |
|  | STRONGERTOGETHER |
|  | PHYSICALDISTANCING |
|  | STAYAPART |
|  | HELPPREVENTTHESPREAD |
|  | SOCIALDISTANCING |
|  | STOPTHESPREAD |
| Federal government officials | PHYSICALDISTANCING |
|  | FLATTENTHECURVE |
|  | ECONOMICRESPONSE |
|  | PLANKTHECURVE |
|  | TOGETHERAPART |
|  | STAYHOMESAVELIVES |
|  | STAYHOME |
|  | STRONGERTOGETHER |
|  | MENTALHEALTH |
|  | SOCIALDISTANCING |

**Appendix G**

Sentiment analysis results that depict the evolution of the public’s sentiments towards the COVID-related Canadian public discourse over time.


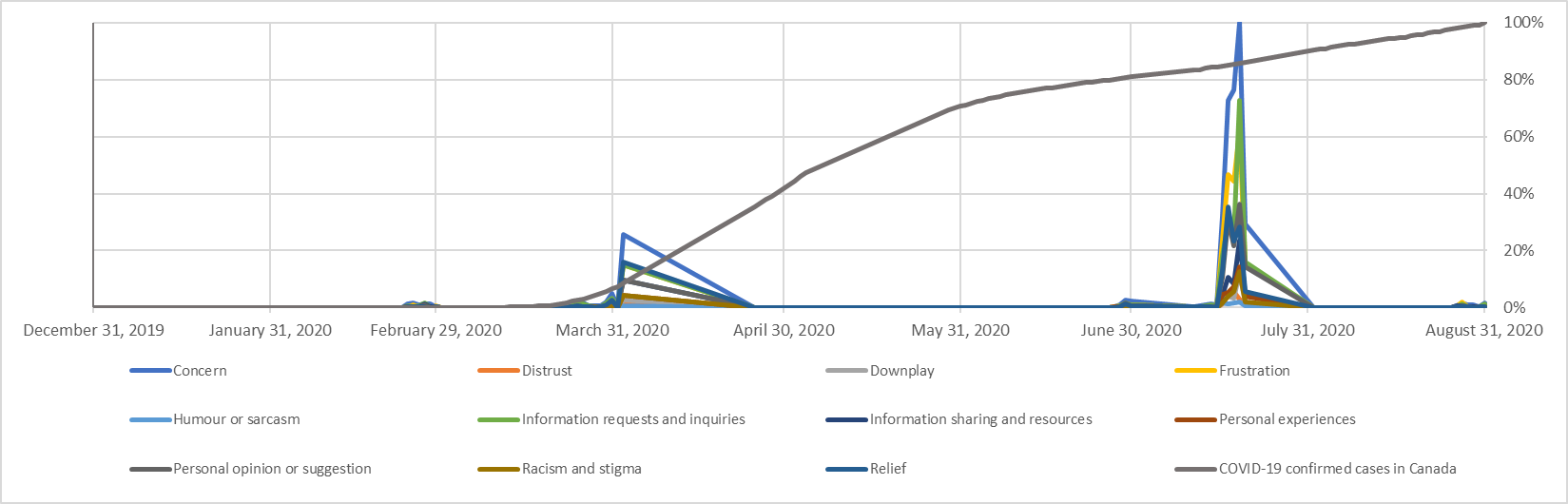


Figure 10: The relative proportion of the sentiments related to the federal government officials of Canada from December 31, 2019, to August 31, 2020. Figure was scaled to the highest peak on July 19, 2020, where 937 (27.19%) of all sentiments were recorded. The peak was assigned a score of 100%.


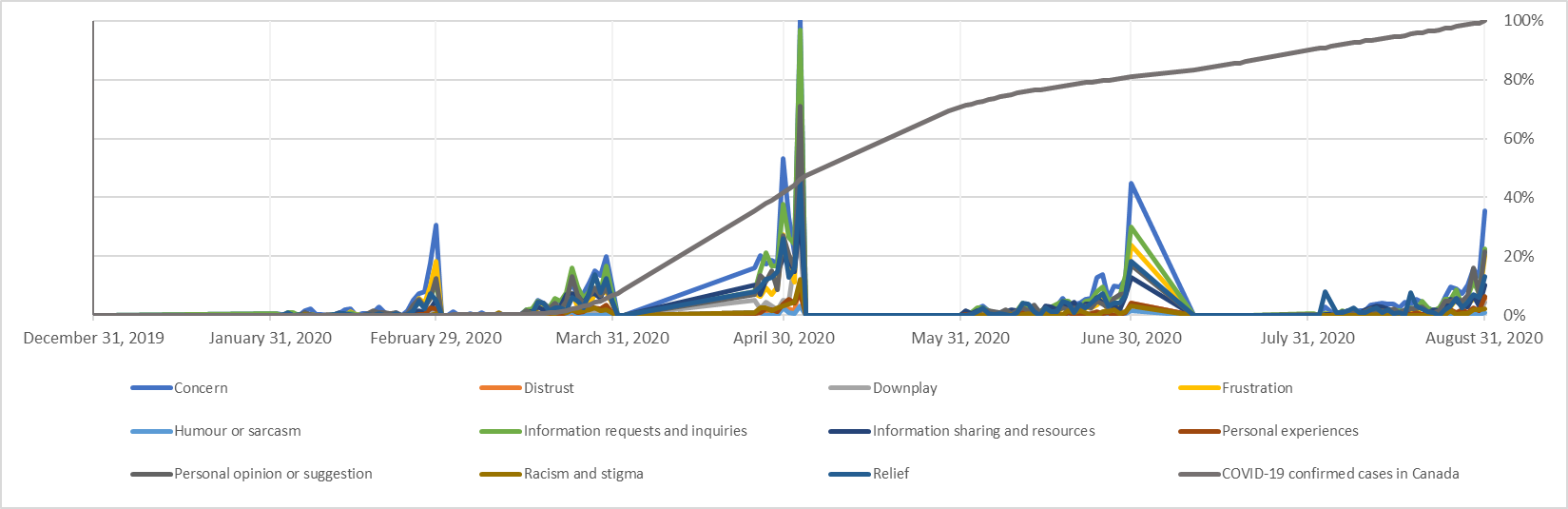


Figure 11: The relative proportion of the sentiments related to the government officials of all the Canadian provinces and territories from December 31, 2019, to August 31, 2020. The Figure was scaled to the highest peak on May 03, 2020, where 435 (20.70%) of all sentiments were recorded. The peak was assigned a score of 100%.


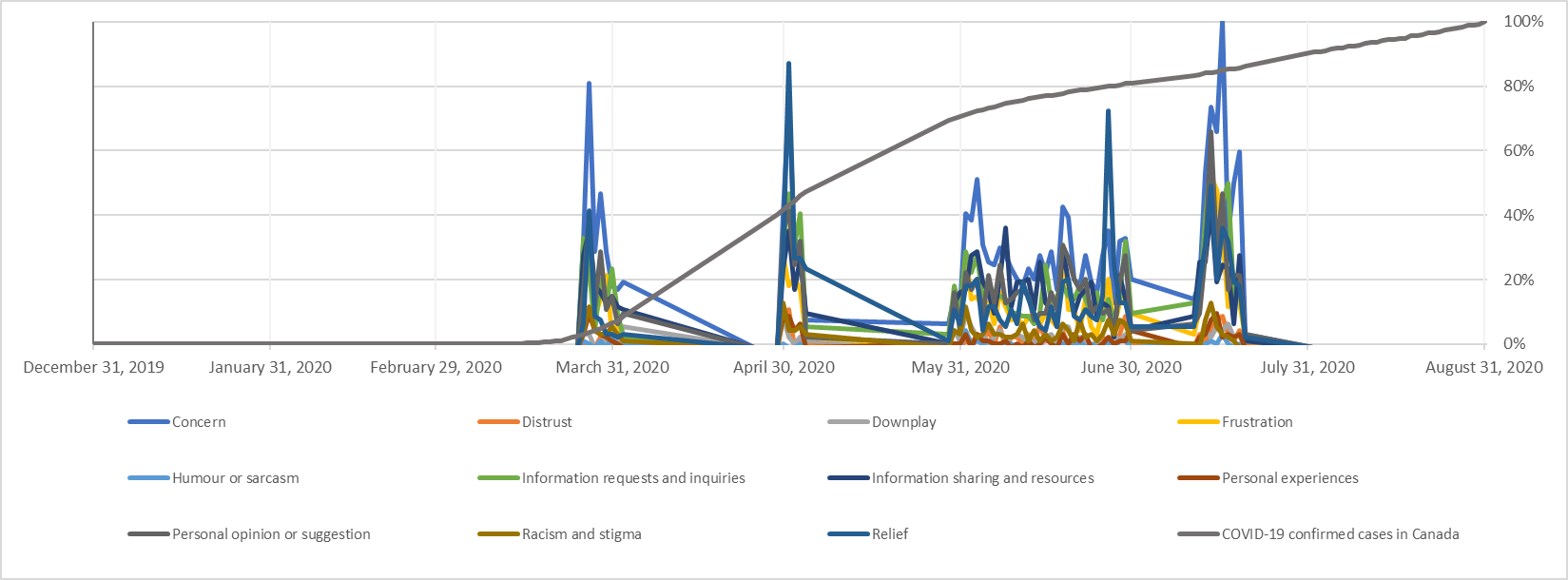


Figure 12: The relative proportion of the sentiments related to the federal health officials of Canada from December 31, 2019, to August 31, 2020. The Figure was scaled to the highest peak on July 16, 2020, where 95 (31.77%) of all sentiments were recorded. The peak was assigned a score of 100%.


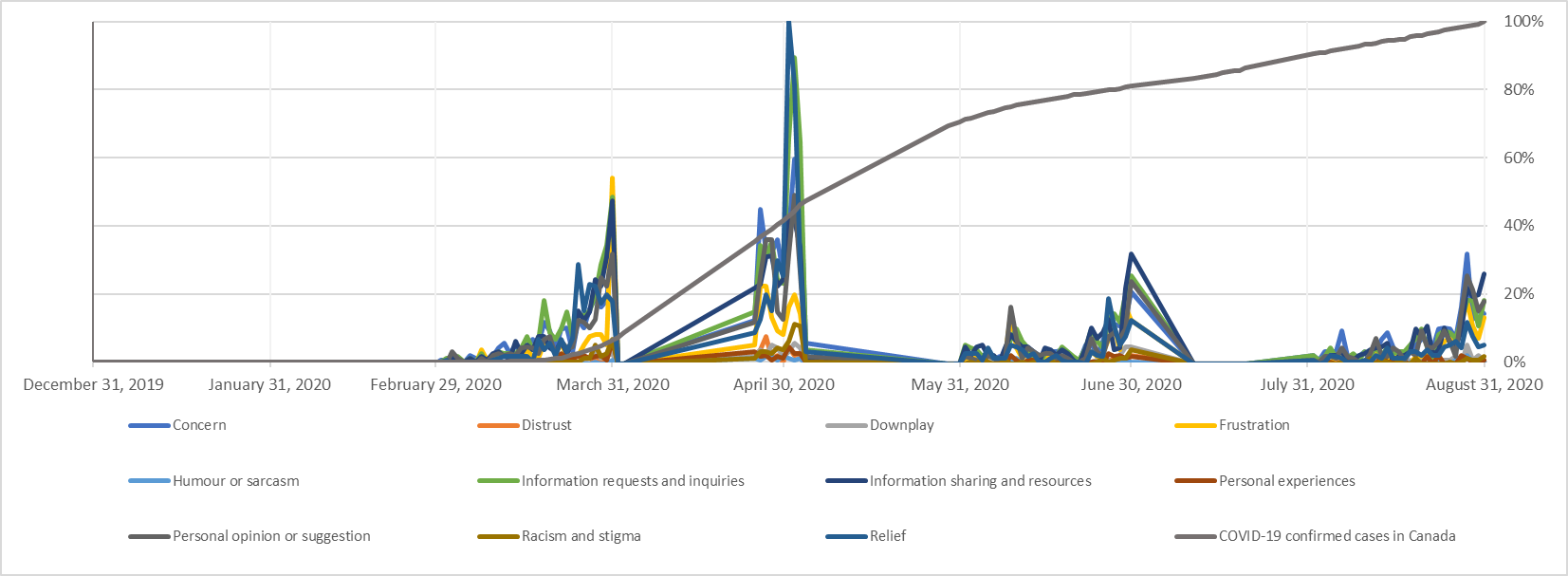


*Figure 13: The relative proportion of the sentiments related to the health officials of all the Canadian provinces and territories from December 31, 2019, to August 31, 2020. The Figure was scaled to the highest peak on May 01, 2020, where 199 (31.53%) of all sentiments were recorded. The peak was assigned a score of 100%.*
